# 中国晚期原发性肺癌诊治专家共识（2016年版）

**DOI:** 10.3779/j.issn.1009-3419.2016.01.01

**Published:** 2016-01-20

**Authors:** 远凯 石, 燕 孙, 金明 于, 翠敏 丁, 子平 王, 长利 王, 东 王, 存德 王, 征 王, 孟昭 王, 修益 支, 铀 卢, 继锋 冯, 云鹏 刘, 晓晴 刘, 巍 刘, 钢 伍, 小梅 李, 凯 李, 恩孝 李, 薇 李, 公琰 陈, 正堂 陈, 萍 余, 宁 吴, 密璐 吴, 文华 肖, 力 张, 沂平 张, 树才 张, 树军 杨, 霞 宋, 冬梅 林, 荣城 罗, 莉 单, 彩存 周, 宗玫 周, 琼 赵, 成平 胡, 毅 胡, 其森 郭, 建华 常, 诚 黄, 瑄 曾, 宝惠 韩, 晓红 韩, 博 郏, 颖 韩, 昱 黄

**Affiliations:** 1 100021 北京，中国医学科学院北京协和医学院肿瘤医院，抗肿瘤分子靶向药物临床研究北京市重点实验室 Cancer Hospital, Chinese Academy of Medical Sciences & Peking Union Medical College, Beijing Key Laboratory of Clinical Study on Anticancer Molecular Targeted Drugs, Beijing 100021, China; 2 250117 济南，山东省肿瘤医院 Shandong Province Cancer Hospital, Ji'nan 250117, China; 3 050000 石家庄，河北医科大学第四医院 Te Fourth Hospital of Hebei Medical University, Shijiazhuang 050000, China; 4 100142 北 京，北京大学肿瘤医院 Beijing Cancer Hospital, Beijing 100142, China; 5 300070 天津，天津医科大学肿瘤医院 Tianjin Medical University Cancer Institute & Hospital, Tianjin 300070, China; 6 400042 重庆，第三军医大学大坪 医院 Daping Hospital, Tird Military Medical University, Chongqing 400042, China; 7 650118 昆明，云南省肿瘤医院 Yunnan Province Cancer Hospital, Kunming 650118, China; 8 100730 北京，卫生部北京医院 Beijing Hospital, Beijing 100730, China; 9 100730 北京，北京协和医院 Peking Union Medical College Hospital, Beijing 100730, China; 10 100053 北京，首都医科大学宣武医院 Beijing Xuanwu Hospital, Capital Medical University, Beijing 100053, China; 11 610041 成都，四川大学华西医院 West China Hospital of Sichuan University, Chengdu 610041, China; 12 210009 南京，江苏省肿瘤医院 Jiangsu Cancer Hospital, Nanjing 210009, China; 13 110001 沈阳，中国医科大学附属第一医院 Te First Hospital of China Medical University, Shenyang 110001, China; 14 100071 北京，中国人民解放军第307医院 Te 307th Hospital of Chinese People's Liberation Army, Beijing 100071, China; 15 430022 武汉，华中科技大 学协和医院 Huazhong University of Science and Technology Union Hospital, Wuhan 430022, China; 16 100853 北京，中国人民解放军总医院 Chinese People's Liberation Army General Hospital, Beijing 100853, China; 17 710061 西安，西安交通大学第一附属医院 Te First Afliated Hospital of Xi'an Jiaotong University, Xi'an 710061, China; 18 130021 长春，吉林大学第一医院 Te First Hospital of Jilin University, Changchun 130021, China; 19 150081 哈尔滨，哈尔滨医科大学附属肿瘤医院 Harbin Medical University Cancer Hospital, Harbin 150081, China; 20 400037 重庆，第三军医大学新桥医院 Xinqiao Hospital of Te Tird Military Medical University, Chongqing 400037, China; 21 610047 成都，四川省肿瘤医院 Sichuan Cancer Hospital, Chengdu 610047, China; 22 100021 北京，中国医学科学院北京协和医学院肿瘤医院 Cancer Hospital, Chinese Academy of Medical Sciences & Peking Union Medical College, Beijing 100021, China; 23 810000 西宁，青海 大学附属医院 Qinghai University Afliated Hospital, Xining 810000, China; 24 100048 北京，中国人民解放军总医院第一附属医院 Te First Afliated Hospital of Chinese People's Liberation Army General Hospital, Beijing 100048, China; 25 310022 杭州，浙江省肿瘤医院 Zhejiang Cancer Hospital, Hangzhou 310022, China; 26 101149 北京，首都医科大学附属北京胸科医院 Beijing Chest Hospital, Capital Medical University, Beijing 101149, China; 27 450008 郑州，河南省肿瘤医院 Henan Province Cancer Hospital, Zhengzhou 450008, China; 28 030013 太原，山西省肿瘤医院 Shanxi Province Cancer Hospital, Taiyuan 030013, China; 29 510515 广州，南方医科大学南方医院 Nanfang Hospital, Nanfang Medical University, Guangzhou 510515, China; 30 830000 乌鲁木齐，新疆医科大学肿瘤医院 Cancer Hospital of Xinjiang Medical University, Urumqi 830000, China; 31 200433 上海，同济大学附属上海市肺科医院 Shanghai Pulmonary Hospital, Tongji University, Shanghai 200433, China; 32 310003 杭州，浙江大学附属第一医院 Te First Afliated Hospital, Zhejiang University, Hangzhou 310003, China; 33 410008 长沙，中南大学湘雅医院 Xiangya Hospital, Central South University, Changsha 410008, China; 34 200032 上海，复旦大学附属肿瘤 医院 Cancer Hospital, Fudan Universitay, Shanghai 200032, China; 35 350014 福州，福建省肿瘤医院 Fujian Cancer Hospital, Fuzhou 350014, China; 36 200030 上海，上海交通大学附属胸科医院 Shanghai Chest Hospital, Shanghai Jiaotong University, Shanghai 200030, China

## 一 概述

原发性肺癌(以下简称肺癌)是我国最常见的恶性肿瘤之一。国家癌症中心2015年发布的数据显示，2006年-2011年我国肺癌5年患病率是130.2(1/10万)。其中男性84.6(1/10万)，居恶性肿瘤第2位。女性45.6(1/10万)，居恶性肿瘤第4位^[1]^。

国际肺癌研究协会(International Association for the Study of Lung Cancer, IASLC)2015年制定了第八版肺癌肿瘤-淋巴结-转移(tumor-node-metastasis, TNM)分期。美国医疗保险监督、流行病学和最终结果(Surveillance, Epidemiology, and End Results, SEER)数据库显示，在初诊时57%的肺癌患者已经发生了远处转移^[2]^，所以晚期患者的治疗是肺癌治疗体系的重要组成部分，也是近年来进展最多的部分。病理诊断是肺癌诊断的金标准，与此同时，近年来肺癌的分子遗传学研究取得了显著进展，基于遗传特征的分子分型使晚期肺癌的治疗步入了个体化分子靶向治疗时代。正是在这样的背景下，2015年世界卫生组织(World Health Organization, WHO)发表了肺肿瘤组织学的新分类^[3]^。与2004年分类相比，其中一项最主要的变化就是在晚期肺癌患者的个体化治疗策略中强调了分子遗传学的作用。

国家卫生和计划生育委员会医政医管局委托中国抗癌协会肿瘤临床化疗专业委员会制定了《中国原发性肺癌诊疗规范(2015年版)》 ^[4]^，但是近些年晚期肺癌的治疗进展迅速，治疗选择显著增多，为进一步提高我国晚期肺癌的诊疗水平，改善晚期肺癌患者的预后，中国医师协会肿瘤医师分会和中国抗癌协会肿瘤临床化疗专业委员会组织全国专家，结合近年来肺癌病理、分子遗传学及诊断和治疗的最新研究成果，制定了《中国晚期原发性肺癌诊疗专家共识(2016年版)》。

## 二 临床表现

晚期肺癌患者可出现刺激性干咳、咯血、胸痛、发热、气促等症状。当肿瘤在胸内蔓延侵及周围组织时，可导致声音嘶哑、上腔静脉阻塞综合征(superior vena caval obstruction syndrome)、霍纳氏综合征(Horner syndrome)、胸腔积液及心包积液等。远处转移至脑、骨、肝、肾上腺及其他器官时，可引起相应器官转移的临床表现。另外，部分患者可出现副肿瘤综合征(paraneoplastic syndromes)，包括库欣综合征(Cushing syndrome)、抗利尿激素分泌异常综合征(syndrome of inappropriate antidiuretic hormone, SIADH)、高钙血症、类癌综合征(carcinoid syndrome)及继发增殖性骨关节病等。

## 三 体格检查

部分晚期肺癌患者可出现杵状指(趾)、男性乳腺增生、皮肤黝黑或皮肌炎和共济失调等征象。体检发现声带麻痹、上腔静脉阻塞综合征、霍纳氏综合征等表现时，需警惕肺癌局部侵犯及转移。出现皮下结节、锁骨上淋巴结肿大等需除外远处转移。

## 四 辅助检查

(一)、实验室检查

1、一般检查

患者在治疗前，应行血常规、肝肾功能和病毒指标等实验室检查，以了解患者的一般状况及是否适于采取相应的治疗措施。进行有创检查或手术治疗的患者，还需进行凝血功能检测。

2、肿瘤标志物(tumor markers, TMs)

肺癌相关的血清肿瘤标志物包括癌胚抗原(carcinoembr yoni c antigen, CEA)、糖类抗原125 (carbohydrate antigen 125, CA125)、糖类抗原153 (carbohydrate antigen 153, CA153)、细胞角蛋白片段19 (cytokeratin fragment, CYFRA21-1)、鳞状上皮细胞抗原(squamous cell carcinoma antigen, SCCA)等，小细胞肺癌(small cell lung cancer, SCLC)具有神经内分泌特点，与促胃泌素释放肽前体(progastrin-releasing peptide, ProGRP)、神经元特异性烯醇化酶(neuron-specific enolase, NSE)、肌酸激酶BB(creatine kinase BB isoenzyme, CK-BB)以及嗜铬蛋白A(CgA)等相关，可作为监测治疗反应和早期复发的辅助指标，联合使用可提高其在临床应用中的敏感度和特异度。

3、血清表皮生长因子受体(epidermal growth factor receptor, EGFR)基因突变检测

与肿瘤组织相比，循环肿瘤DNA(circulating tumor DNA, ctDNA)中*EGFR*基因突变检测具有高度特异性(IPA SS、IFUM和IGNITE研究^[5-7]^中的特异性分别为100%、99.8%和97.2%)，但敏感度相对较低(分别为43.1%、65.7%和49.6%)；这可能与肿瘤分期、血液标本的处理、检测方法差异等相关。欧洲药品管理局2014年9月25日批准当难以获取肿瘤组织样本时，可采用外周血ctDNA作为补充标本评估*EGFR*基因突变状态，以明确可能从吉非替尼治疗中获益的非小细胞肺癌(non-small cell lung cancer, NSCLC)患者。中国食品与药品监督管理局(Chinese Food and Drug Administration, CFDA)于2015年2月13日批准吉非替尼说明书进行更新，在推荐所有NSCLC患者的肿瘤组织都应进行*EGFR*基因突变检测基础上，补充了如果肿瘤标本不可评估，可使用从血液(血浆)标本中获得的ctDNA进行评估，以尽可能明确最可能从吉非替尼治疗中受益的NSCLC患者。因此，血液(血浆)标本检测ctDNA评估*EGFR*基因突变状态是选择EGFR-酪氨酸激酶抑制剂(tyrosine kinase inhibitors, TKIs)治疗的补充手段。

(二)、影像学检查

肺癌的影像检查方法主要包括：X线胸片、计算机断层扫描(computed tomography, CT)、磁共振成像(magnetic resonance imaging, MRI)、超声、核素显像、正电子发射计算机断层扫描(positron emission computed tomography, PET) -CT等。主要用于晚期肺癌诊断、分期、再分期、疗效监测及预后评估等。

1、胸部X线检查：X线胸片是发现晚期肺癌的重要手段，也是晚期肺癌治疗前后基本的影像学检查方法。

2、胸部CT检查：胸部CT对于晚期肺癌诊断、分期、疗效评价及治疗后随诊具有重要意义，是肺癌最重要和最常用的影像学检查方法。建议用螺旋CT以≤10 mm的层厚扫描，无禁忌症的患者一般应予静脉对比增强，以区别肿瘤病灶与邻近的血管和软组织。如用于疗效评估，原则上要求最小病灶不应小于2倍层厚扫描；且每次必须在相同的窗位测量病灶。

3、MRI检查：MRI特别适用于判定脑、脊髓有无转移。另外，MRI检查可用于判定胸壁或纵隔是否受侵；显示肺上沟瘤与臂丛神经及血管的关系。对于禁忌注射碘造影剂的患者，MRI是观察纵隔、肺门大血管受侵情况及淋巴结肿大的首选检查方法。扫描要求与CT检查相同。

4、超声检查：主要用于发现腹部实性重要器官以及腹腔、腹膜后淋巴结有无转移，也用于双侧锁骨上淋巴结的检查；超声还常用于胸腔积液及心包积液抽取时的定位。

5、放射性核素骨扫描检查：是用于判断肺癌骨转移的常规检查。当骨扫描检查提示骨可疑转移时，对可疑部位进行MRI、CT或PET-CT等检查验证。

6、PET-CT检查：是肺癌诊断、分期与再分期、疗效评价和预后评估的最佳方法。

(三)、内窥镜检查

内窥镜检查可获取细胞学和组织学诊断，主要包括支气管镜检查、经支气管针吸活检术(transbronchial needle aspiration, TBNA)、超声支气管镜引导的TBNA (endobronchial ultrasound-guided TBNA, EBUS-TBNA)、经支气管肺活检术(transbronchial lung biopsy, TBLB)、纵隔镜检查及胸腔镜检查。

(四)、其他检查技术

痰细胞学检查、经胸壁肺内肿物穿刺针吸活检术(transthoracic needle aspiration, TNA)、胸腔穿刺术、胸膜活检术、浅表淋巴结及皮下转移结节活检术都是晚期肺癌诊断的重要方法。

## 五 病理诊断

(一)、标本固定标准

使用10%中性缓冲福尔马林固定液，避免使用含有重金属的固定液，固定液量应为所固定标本体积≥10倍，常温固定。标本从离体到固定时间不宜超过30 min。活检标本直接放入固定液，支气管镜活检标本的固定时间为6 h-24 h，手术切除标本的固定时间为12 h-48 h。

获取的不同类型细胞学标本制片固定应采用95%乙醇固定液，时间不宜少于15 min，或采用非妇科液基细胞学固定液，固定时间和方法可按说明书进行操作；所有细胞学标本应尽量制作福尔马林固定石蜡包埋(formalin-fxed and parrfn-enbedded, FFPE)细胞学蜡块。将细胞学标本离心沉淀置于包埋盒中，后续操作同组织学标本制作蜡块流程。

(二)、标本大体描述及取材要求

活检标本核对无误后将送检组织全部取材。

(三)、取材后标本处理原则和保留时限

取材剩余组织保存在标准固定液中，并始终保持充分的固定液量和福尔马林浓度，以备在病理诊断报告签发后接到临床反馈信息时复查大体标本或补充取材。剩余标本处理的时限建议在病理诊断报告签发1个月后，未接到临床反馈信息，未发生因外院会诊意见分歧而要求复审等情形后，由医院自行处理。

(四)、组织病理诊断

小活检组织标本肺癌病理诊断主要解决有无肿瘤及肿瘤类型，对于形态学不典型的病例或晚期不能手术的患者病理诊断需结合免疫组化(immunohistochemistry, IHC)染色尽可能进行亚型分类，尽量避免使用非特殊类型(NSCLC-NOS)的诊断。

(五)、病理报告内容

临床信息包括姓名、性别、年龄、病历号、送检科室和医生、病变部位、活检方式或手术方式、相关肿瘤史和治疗史。大体描述内容包括标本类型、肿瘤大小、与支气管或胸膜的关系、其他伴随病变或多发病变、切缘。诊断内容包括肿瘤部位、组织学亚型。

(六)、IHC和特殊染色

腺癌与鳞状细胞癌鉴别的IHC标记物宜选用TF-1、Napsin-A、p63、P40和CK5/CK6；神经内分泌肿瘤标记物宜选用CD56、Syn、CgA、Ki-67和TTF-1，在具有神经内分泌形态学特征基础上，至少有一种神经内分泌标记物明确阳性，阳性细胞数应> 10%肿瘤细胞量才可诊断神经内分泌肿瘤；细胞内粘液物质的鉴别宜进行粘卡、AB-PAS特殊染色；可疑累及胸膜时应进行弹力纤维特殊染色确认。

(七)、分子病理检测^[8]^

对于晚期NSCLC、腺癌或含腺癌成分的其他类型肺癌，应在诊断的同时常规进行EGFR基因突变和间变性淋巴瘤激酶(anaplastic lymphoma kinase, *ALK*)融合基因检测。如有必要可进行*c-ros*原癌基因1酪氨酸激酶(c-ros oncogene 1 receptor tyrosine kinase, *ROS1*)基因及*RET*基因融合，*K-RAS*基因和*BRAF*基因V600E突变、人类表皮生长因子受体2(human epidermal growth factor receptor-2, *HER2*)基因扩增、*MET*基因高水平扩增及*MET*基因14号外显子跳跃缺失突变检测。

1、*EGFR*基因突变检测

推荐所有病理诊断为肺腺癌、含有腺癌成分和具有腺癌分化的NSCLC患者进行*EGFR*基因突变检测，建议对于小活检标本诊断的或不吸烟的鳞癌患者也进行检测。

*EGFR*基因突变检测的标本和处理方法：手术切除和活检的组织标本是最常见的用于*EGFR*基因突变检测的标本类型，建议优先选择组织标本进行检测，规范处理的组织标本可以满足检测要求。原发灶和转移灶的组织标本均可用于*EGFR*基因突变检测，细胞学标本也可以用于检测。

应规范不同标本的处理方法，组织标本的固定应使用4%中性缓冲甲醛固定液或10%中性缓冲福尔马林固定液，避免使用酸性及含有重金属离子的固定液。活检组织标本一般固定6 h-12 h，手术切除标本需固定6 h-48 h。

肿瘤组织切片应由病理医师审阅复核，评估肿瘤细胞含量，必要时在显微镜下定位标出肿瘤组织区域进行人工切割刮取组织，以保证有足量的肿瘤细胞提取DNA。对于肿瘤细胞数量不达标的样本应重新采集。

*EGFR*基因突变检测方法：目前，检测*EGFR*基因突变最常用的方法是直接测序法和扩增阻遏突变系统(Amplifcation Refractory Mutation System, ARMS)。建议使用权威机构批准上市的*EGFR*基因突变检测试剂盒。

检测信息应包括患者的基本个人信息、病历号、病理诊断、标本类型、肿瘤细胞含量(如肿瘤细胞数量或百分比)、检测方法、检测结果，同时标明标本接收日期和报告日期，由检测员和另一位有经验的医师审核并出具报告。检测结果中*EGFR*基因突变类型应用国际通用的人类基因组变异协会(Human Genome Variation Society, HGVS)命名法则命名。

2、*ALK*融合基因检测

推荐所有病理诊断为肺腺癌、含有腺癌成分和具有腺癌分化的NSCLC患者进行*ALK*融合基因检测。

*ALK*融合基因检测的标本类型：肿瘤原发或转移部位的组织或细胞学标本均可进行*ALK*融合基因检测，标本处理的要求与*EGFR*基因突变检测相同。

无论采用哪种标本类型，均应保证足够的肿瘤细胞，尽量剔除非肿瘤组织和细胞。石蜡组织切片厚度一般为(5±1) μm。

*ALK*融合基因检测方法：目前用于*ALK*融合基因的检测方法主要有荧光原位杂交(fluorescence *in situ* hybridization, FISH)、IHC和逆转录聚合酶链反应(reverse transcriptase-polymerase chain reaction, RT-PCR)等。FISH能特异和灵敏地检测出*ALK*融合基因，是目前检测*ALK*融合基因的经典方法，在克唑替尼上市时被美国食品药品管理局(Food and Drug Administration, FDA)批准为EML4-ALK阳性NSCLC的伴随诊断方法。FISH探针包括分离探针和融合探针，分离探针与克唑替尼疗效显示较好的相关性。RT-PCR能够灵敏地检测出已知类型的融合基因。CFDA批准的IHC技术平台与FISH具有高度的检测一致性。

分离探针标记的FISH技术、经权威机构批准的RT-PCR及IHC技术平台均可用于*ALK*融合基因的检测，其他IHC检测平台可作为*ALK*融合基因的初筛手段，建议以FISH或RT-PCR方法确认。

在检测报告中需要注明检测方法、检测平台，FISH法需要注明肿瘤细胞数及阳性细胞比例。对患者和标本等基本信息的要求同*EGFR*基因检测部分。

*EGFR*基因突变和*ALK*融合基因检测时标本的处理和质量控制均应由有经验的病理科医师负责，所有标本均应在尽量短的时间内进行检测，在进行切片时应有措施避免不同病例病理组织间的交叉污染。

3、EGFR-TKI耐药后的分子病理检测

EGFR-TKI治疗失败的患者在条件允许的情况下应再取肿瘤组织活检，明确病变组织类型，如果为NSCLC，建议进行T790M突变、*MET*基因扩增、*HER2*基因扩增、*PIK3CA*突变、*BRAF*基因V600E突变、*ERK*扩增等检测。

## 六 分期

(一)、NSCLC

目前晚期NSCLC的分期采用IASLC2009年第七版分期标准或2015年第八版分期标准。第七版分期标准中M1a包括胸腔/心包积液、对侧或双侧肺结节或胸膜结节；M1b指远处转移^[9]^。第八版分期标准中M1a包括胸腔/心包积液、对侧或双侧肺结节或胸膜结节；M1b包括单个器官的孤立转移；M1c包括单个器官的多处转移或多个器官的多处转移^[10]^。

(二)、SCLC

目前广泛期SCLC的分期可采用美国退伍军人肺癌协会(Veterans Administration Lung Study Group, VALG)提出的局限期(limited disease, LD)和广泛期(extensive disease, ED)分期方法。广泛期为病变超出同一侧胸腔，包括恶性胸腔积液、心包积液及远处转移^[11]^。近年来IASLC建议SCLC同时采用NSCLC的TNM分期，广泛期患者均为Ⅳ期(T_any_，N_any_，M1a/M1b；包括T3、T4多发肺结节)。

七、治疗

(一)、治疗原则

晚期肺癌应采用以全身治疗为主的综合治疗，根据患者的病理类型、分子遗传学特征以及患者的机体状态制定个体化的治疗策略，以期最大程度地延长患者生存时间、控制疾病进展程度、提高生活质量。

1、晚期NSCLC的治疗

晚期NSCLC的治疗原则是以全身治疗为主的综合治疗。在一线治疗前应首先获取肿瘤组织，明确病理分型和分子遗传学特征，根据检测结果决定治疗方案。

晚期NSCLC患者的全身治疗：

(1)、*EGFR*基因敏感突变并且不存在耐药基因的晚期NSCLC患者推荐EGFR-TKIs一线治疗，*ALK*融合基因阳性患者推荐克唑替尼一线治疗。

(2)、*EGFR*基因敏感突变和*ALK*融合基因阴性或突变状况未知的晚期NSCLC患者，如果美国东部肿瘤协作组(Eastern Cooperative Oncology Group, EOCG)体力状况(performance status, PS)评分为0分-1分，应当尽早开始含铂两药方案的全身化疗(推荐化疗方案见表 1)。对不适合铂类药物治疗的患者，可考虑非铂类两药联合方案化疗。对于合适的患者，可以考虑联合血管生成抑制剂治疗。

**1:** 常用的非小细胞肺癌一线化疗方案

化疗方案	剂量(mg/m^2^)	用药时间	时间及周期
NP:长春瑞滨	25	d1、d8	q21d×4-6
顺铂	80	d1	
TP:紫杉醇	135-175	d1	q21d×4-6
顺铂	75	d1	
或卡铂	曲线下面积=5-6	d1	
GP:吉西他滨	1, 250	d1、d8	q21d×4-6
顺铂	75	d1	
或卡铂	曲线下面积=5-6	d1
DP:多西他赛	75	d1	q21d×4-6
顺铂	75	d1	
或卡铂	曲线下面积=5-6	d1	
PC:培美曲塞	500	d1	q21d×4-6
顺铂	75	d1	
或卡铂	曲线下面积=5-6	d1	
SP:替吉奥	40 mg/m^2^po bid	d1-d21	q35d×6
顺铂	60	d8	
表1化疗方案中药物的推荐剂量仅供参考，由于每位患者存在个体差异，具体药物剂量和用药时间可根据患者体质及药物不良反应进行调整。			

(3)、ECOG PS评分为2分的晚期NSCLC患者应给予单药化疗，ECOG PS评分≥3分的患者不建议使用细胞毒类药物化疗，建议采用最佳支持治疗。

(4)、二线治疗可选择的药物包括多西紫杉醇、培美曲塞和EGFR-TKIs。*EGFR*基因敏感突变且不合并耐药突变的患者，如果一线和维持治疗时没有应用EGFRTKIs，二线治疗时应优先应用EGFR-TKIs；对于*EGFR*基因敏感突变阴性的患者，应优先考虑化疗。

在全身治疗基础上针对具体的局部情况，可以选择恰当的局部治疗方法以求改善症状、提高生活质量。

2、广泛期SCLC的治疗

广泛期SCLC应采用化疗为主的综合治疗。一线治疗推荐EP方案(依托泊苷联合顺铂)、EC方案(依托泊苷联合卡铂)、IP方案(伊立替康联合顺铂)、IC方案(伊立替康联合卡铂)。化疗有效患者可考虑行预防性全脑照射(prophylactic cranial irradiation, PCI)治疗。如果化疗有效者，且远处转移病灶控制，一般情况尚好者可行胸部病变放疗。

(二)、内科治疗

1、晚期NSCLC的化疗

(1)、一线化疗

在我国，长春瑞滨、吉西他滨、多西他赛、紫杉醇联合铂类是最常见的含铂两药联合化疗方案^[12]^。对于非鳞癌NSCLC，培美曲塞联合顺铂方案疗效明显优于吉西他滨联合顺铂方案，并且耐受性更好。2014年5月4日，CFDA批准培美曲塞联合顺铂应用于局部晚期或转移性非鳞癌NSCLC患者的治疗。

替吉奥(S-1)联合顺铂或卡铂是一个新的一线治疗晚期NSCLC的化疗方案。我国进行的SC-103试验结果显示，S-1联合顺铂(SP)组一线治疗晚期NSCLC的无进展生存期(progression-free survival, PFS)和总生存期(overall survival, OS)非劣效于多西他赛联合顺铂(DP)组。SP组3度/4度中性粒细胞减少性发热及中性粒细胞减少的发生率明显低于DP组^[13]^，但目前我国CFDA尚未批准该药应用于晚期NSCLC患者的治疗。

紫杉醇(白蛋白结合型)(paclitaxel, Abraxane)联合卡铂是另一个新的一线治疗晚期NSCLC的有效方案。Ⅲ期临床试验结果显示，对于晚期肺鳞癌患者紫杉醇(白蛋白结合型)联合卡铂方案的总有效率明显高于紫杉醇联合卡铂方案，而对于非鳞NSCLC患者两方案的总有效率相似。亚组分析显示，对于年龄大于70岁的老年患者，与紫杉醇联合卡铂方案相比，紫杉醇(白蛋白结合型)联合卡铂方案显著提高了OS。除此之外，紫杉醇(白蛋白结合型)严重周围神经毒性及中性粒细胞减少的发生率明显低于紫杉醇组^[14]^。因此，2012年10月11日美国FDA批准紫杉醇(白蛋白结合型)与卡铂联合应用于晚期NSCLC患者的治疗，但目前我国CFDA尚未批准该药用于晚期NSCLC的治疗。

目前可用于晚期NSCLC一线化疗的药物见表 1。

(2)、维持治疗

对一线化疗达到疾病控制[完全缓解(complete remission, CR) +部分缓解(partial remission, PR) +稳定(stable disease, SD)]的晚期NSCLC患者，可选择维持治疗。按照是否沿用一线化疗方案中的药物，将维持治疗分为同药维持治疗和换药维持治疗两种方式。培美曲塞可以用于非鳞癌NSCLC的同药维持治疗，另外，吉西他滨也可以用于NSCLC的同药维持治疗，换药维持治疗的药物有多西他赛和用于非鳞癌NSCLC的培美曲塞。培美曲塞用于晚期NSCLC换药维持治疗的研究显示，一线含铂方案化疗后培美曲塞维持治疗可延长PFS及OS，晚期非鳞癌NSCLC患者培美曲塞联合顺铂化疗后培美曲塞同药维持治疗较安慰剂明显延长OS^[15]^。多西他赛用于维持治疗的研究仅显示PFS获益，并未获得OS的延长^[16]^。

(3)、二线/三线化疗

二线化疗可选择的化疗药物包括多西他赛和用于非鳞癌NSCLC的培美曲塞。三线治疗可参加临床试验或给予最佳支持治疗。

2、广泛期SCLC的化疗

由于SCLC的生物学特性与其他组织学类型不同，诊断时广泛期占2/3。化疗是广泛期SCLC最主要的治疗手段，是广泛期SCLC患者的一线标准治疗。对于ECOG PS评分0分-2分者，推荐的一线化疗方案有EP方案、EC方案、IP方案或IC方案。临床试验已证实对于未经治疗的广泛期SCLC患者，IP方案在疗效上不劣于EP方案^[17]^。广泛期SCLC、ECOG PS评分3分-4分者，可在最佳支持治疗的基础上，根据患者的肿瘤情况、机体状况、患者及家属的意愿等进行综合分析，权衡利弊，谨慎地选择治疗方案，可能的选择包括单药化疗、减少剂量的联合化疗、必要时联合局部放疗等。一线化疗后如果全身播散病灶少、治疗后疾病控制良好、ECOG PS评分为0分-2分者，经选择的患者可进行胸部放疗；一线治疗达CR、ECOG PS评分为0分-2分者，可考虑PCI。

目前常用的SCLC化疗方案见表 2。

**2:** 常用的小细胞肺癌一线化疗方案

化疗方案	剂量(mg/m^2^)	用药时间	时间及周期
EP:依托泊苷	100	d1-d3	q21d×4-6
顺铂	80	d1	
或卡铂	曲线下面积=5-6	d1	
或依托泊苷	120	d1-d3	q21d×4-6
顺铂	60	d1	
IP:伊立替康	60	d1、d8、d15	q28d×4-6
顺铂	60	d1	
或伊立替康	65	d1、d8	q21d×4-6
顺铂	30	d1、d8	
或伊立替康	50	d1、d8、d15	q28d×4-6
卡铂	曲线下面=5-6	d1	
表2化疗方案中药物的推荐剂量仅供参考，由于每位患者存在个体差异，具体药物剂量和用药时间可根据患者体质及药物不良反应进行调整。			

一线化疗后或化疗期间出现疾病进展的广泛期SCLC患者，选择二线化疗或参加临床试验。临床上将复发患者分为3类：①难治性复发：一线化疗过程中疾病进展；②耐药复发：一线化疗结束后3个月内疾病进展；③敏感复发：一线化疗结束3个月以后疾病进展。二线化疗的疗效与患者对一线化疗的反应及从一线化疗到疾病复发的时间有关。总体上，二线化疗的有效率和缓解期均不如一线化疗，一线化疗有效者病情进展后再次化疗更可能获益，难治或耐药复发患者对大多数药物的疗效差，有效率≤10%，敏感复发者的预期有效率约为25%。3个月内疾病复发进展的患者推荐参加临床试验。3个月-6个月内复发者推荐拓扑替康、伊立替康、吉西他滨或紫杉醇治疗。6个月后疾病进展者可选择初始治疗的化疗方案。

3、抗血管生成药物治疗

(1)、重组人血管内皮抑制素(rh-endostatin，恩度)：Ⅲ期临床试验的结果显示，在长春瑞滨联合顺铂方案一线化疗的基础上联合恩度，能显著延长晚期N S CL C患者的有效率和中位至疾病进展时间(time to progression, TP)，两组之间毒副反应无显著差异。2006年7月24日CFDA批准恩度与化疗联合用于治疗Ⅲ期/Ⅳ期NSCLC患者^[18]^。

(2)、贝伐珠单抗(Bevacizumab)：ECOG 4599研究^[19]^和BEYOND研究^[20]^的结果均显示，在紫杉醇联合卡铂方案一线化疗的基础上，联合贝伐珠单抗化疗之后再用贝伐珠单抗进行维持治疗，能显著延长晚期非鳞癌NSCLC的OS和PFS。AVAPERL研究^[21]^结果显示，培美曲塞联合顺铂和贝伐珠单抗化疗4个周期后用培美曲塞联合贝伐珠单抗两药维持较贝伐珠单抗单药维持更能明显延长PFS。2015年7月9日CFDA批准贝伐珠单抗联合卡铂和紫杉醇用于不可切除的晚期、转移性或复发性非鳞癌NSCLC患者的一线治疗。

4、EGFR-TKIs

EGFR是目前肺癌研究最充分的分子靶点。肺腺癌患者*EGFR*基因突变率在白种人群约为17%^[22]^，PIONEER研究显示在亚裔和我国人群分别为51.4%^[23]^和50.2%^[24]^。

(1)、一线治疗

IPASS、First-SIGNAL、WJTOG 3405、NEJGSG002、OPTIMAL、EURTAC、LUX-Lung 3、LUX-Lung 6研究^[25-32]^均显示，对于*EGFR*基因敏感突变的晚期NSCLC患者，与标准的一线化疗方案相比，EGFR-TKIs(吉非替尼、厄洛替尼、阿法替尼)在PFS、生活质量以及耐受性方面都具有显著的优势。一项全部纳入中国患者的Ⅳ期临床研究^[33]^显示，埃克替尼一线治疗*EGFR*敏感突变晚期NSCLC患者的ORR为56.3%。因此EGFR-TKIs是*EGFR*基因敏感突变晚期NSCLC患者一线治疗的标准选择。吉非替尼和埃克替尼分别于2011年2月22日和2014年11月13日获得CFDA批准用于一线治疗*EGFR*基因敏感突变的晚期NSCLC患者。

(2)、维持治疗

SATURN、INFORM、EORTC08021研究比较了EGFR-TKIs(吉非替尼、厄洛替尼)与安慰剂对一线含铂两药方案化疗后疾病控制患者维持治疗的疗效，结果显示EGFR-TKIs组中位PFS优于对照组。*EGFR*基因突变状态与临床疗效关系的回顾性研究也进一步证实*EGFR*基因突变患者EGFR-TKIs维持治疗后PFS延长^[34]^。因此对于*EGFR*基因敏感突变的晚期NSCLC患者，如果一线化疗后病情没有进展，即疗效评价为CR+PR+SD者，可以选择EGFR-TKIs进行维持治疗。

(3)、二线/三线治疗

BR21和INTEREST的研究^[35, 36]^结果确立了EGFR-TKIs厄洛替尼和吉非替尼在晚期NSCLC二线/三线治疗中的地位。ICOGEN研究将埃克替尼与吉非替尼进行头对头比较，结果显示埃克替尼组患者PFS及OS均非劣效于吉非替尼组，但是埃克替尼的毒副反应更低。亚组分析结果显示，埃克替尼或吉非替尼对于*EGFR*基因敏感突变患者的PFS和OS显著优于野生型患者^[37]^。因此*EGFR*基因敏感突变的患者，如果一线和维持治疗时没有应用EGFRTKIs，二线治疗时应优先应用EGFR-TKIs。对于*EGFR*基因敏感突变阴性的患者，则应优先考虑化疗^[38]^。三线药物治疗可选择EGFR-TKIs或参加临床试验。

其他潜在的治疗靶点，包括ROS1、HER2、BRAF V600E、cMET等，目前临床研究正在进行当中，鼓励患者参加相应的临床试验。

(4)、耐药后治疗

EGFR-TKIs获得性耐药的机制复杂，包括*EGFR*基因T790M点突变、*MET*基因扩增、磷脂酰肌醇-3-激酶(phosphatidylinositol-3-kinase, *PI3K*)基因突变、*EGFR*基因扩增以及转变为SCLC等，其中约50%是由于T790M突变引起的^[39]^。但仍有部分患者的耐药机制尚不清楚，因此有条件的患者在疾病进展时应再次进行肿瘤组织活检，并进行病理和相关的基因检测以明确耐药的性质。第三代EGFR-TKI Osimertinib(AZD9291)是一种强效口服不可逆的EGFR-TKI，可抑制*EGFR*敏感突变和T790M耐药突变。Osimertinib针对既往接受过EGFR-TKI治疗并进展的亚裔和西方晚期NSCLC患者的Ⅰ期临床试验显示了其良好的疗效和安全性^[40]^。2015年11月13日美国FDA有条件批准Osimertinib上市，治疗既往EGFR-TKIs治疗后疾病进展的T790M突变肺癌患者。针对其他耐药机制治疗的研究正在进行中。

5、ALK-TKIs

*ALK*融合基因是肺癌领域发现的另一个重要的治疗靶点。在NSCLC患者中，*ALK*融合基因阳性的发生率约为5%^[41]^。中国NSCLC患者*ALK*融合基因的阳性率为3%-11%^[42, 43]^。

克唑替尼是一种口服的ALK-TKIs。PROFILE1001、PROFILE1005、PROFILE1007、PROFILE1014和PROFILE1029研究^[44-47]^结果均显示了克唑替尼对于*ALK*融合基因阳性晚期NSCLC患者良好的疗效和安全性。2013年1月22日CFDA批准克唑替尼用于ALK阳性晚期NSCLC患者的治疗。

克唑替尼耐药后的治疗：对于克唑替尼治疗后进展的患者，可选择的新型ALK-TKIs包括色瑞替尼(Ceritinib, LDK378)和Alecensa(Alectinib)。Ⅰ期临床研究结果显示，色瑞替尼对于无论既往是否接受过克唑替尼治疗的*ALK*融合基因阳性晚期NSCLC患者都具有很好的疗效和安全性^[48]^。2014年4月29日美国FDA批准色瑞替尼上市，用于克唑替尼耐药的ALK阳性晚期NSCLC的治疗。Ⅱ期临床研究提示，Alecensa对于接受过克唑替尼治疗的*ALK*融合基因阳性的晚期NSCLC患者同样具有很好的疗效。尤其对于脑转移病灶，局部控制率(disease control rate, DCR)可高达83%^[49]^。2015年12月12日美国FDA批准Alecensa上市，用于克唑替尼耐药的ALK阳性晚期NSCLC的治疗。

在NSCLC患者中检测*EGFR*和*ALK*基因状态具有重要的临床意义，美国、欧洲的权威学术机构均已制订出各自的检测和治疗指南。为了提高我国在该领域诊断和治疗的规范化水平，中国医师协会肿瘤医师分会和中国抗癌协会肿瘤临床化疗专业委员会2013年组织专家制订了《中国*EGFR*基因突变和*ALK*融合基因阳性NSCLC诊断治疗指南》，并于2014年和2015年分别予以更新。

6、针对其他靶点的治疗

一项克唑替尼治疗*ROS1*基因重排阳性晚期NSCLC患者的研究结果显示，应用克唑替尼治疗的患者ORR可达72%^[50]^。针对*MET*基因的扩增或14号外显子跳跃性突变、*RET*基因的重排、*HER2*基因扩增和*BRAF*基因V600E突变等靶向治疗的研究正在进行中。

7、免疫治疗

程序性死亡因子-1(programmed death-1, PD-1)是一种负性共刺激分子，与程序性死亡因子配体(programmed death-legand 1, PD-L1)结合后诱导T细胞凋亡，抑制T细胞活化和增殖。抗PD-1抗体Nivolumab (OPDⅣO)和Pembrolizumab(Key tr uda)与T细胞的PD-1受体结合可以阻断PD-1对T细胞的抑制作用，从而激活杀瘤效应。CheckMate017研究^[51]^结果显示，Nivolumab在既往治疗过的晚期肺鳞癌患者中与多西他赛相比可取得生存获益，获益与PD -L1表达无关。CheckMate057研究^[52]^结果显示，Nivolumab在既往治疗过的晚期肺非鳞癌患者中与多西他赛相比同样可取得生存获益，PD-L1的表达能预测Nivolumab的疗效。Nivolumab于2015年3月4日被美国FDA批准用于既往治疗失败的晚期肺鳞癌的治疗。在KEYNOTE-001研究^[53]^中，Pembrolizumab单药治疗既往治疗失败的晚期NSCLC患者显示出很好的疗效，PD-L1的表达能预测Pembrolizumab的疗效。2015年10月2日，美国FDA加速批准Pembrolizumab用于治疗既往治疗失败且PD -L1蛋白表达阳性的晚期NSCLC患者。Pembrolizumab被批准与伴随诊断检测PD-L1 IHC 22C3试剂盒一起使用，这是首个旨在检测NSCLC PD-L1表达的检测方法。

(三)、外科治疗

晚期NSCLC化疗或靶向治疗效果好的患者，残存病灶可考虑手术切除。对于孤立性转移的晚期NSCLC患者，应采取适当的有针对性的治疗措施。部分有单发对侧肺转移、单发脑或肾上腺转移的晚期NSCLC患者也可行手术治疗。单发性脑转移患者可能会从手术治疗中获益，术后可行全脑放疗(whole brain radiotherapy, WBRT)或立体定向放射外科(stereotactic radiosurgery, SRS)治疗。对于有孤立性肾上腺转移而肺部病变又可切除的NSCLC患者，肾上腺病变也可以考虑切除。

(四)、放射治疗

晚期肺癌放射治疗主要包括姑息放疗和预防性放疗等。姑息性放疗适用于对晚期肺癌原发灶和转移灶的减症治疗，以减轻局部压迫症状、骨转移导致的疼痛以及脑转移导致的神经症状等。PCI适用于全身治疗有效的SCLC患者行PCI可降低广泛期SCLC脑转移发生的风险。对于有广泛转移的晚期NSCLC患者，当患者全身治疗获益明显时，可以考虑采用立体定向放射治疗(stereotactic body radiation therapy, SBRT)治疗残存的原发灶和(或)寡转移灶，争取获得潜在的根治效果。对于广泛期SCLC患者，远处转移灶经化疗控制后加用胸部放疗可以提高肿瘤控制率，延长生存期。

(五)、支持和姑息治疗

支持和姑息治疗目的在于缓解症状、减轻痛苦、改善生活质量、提高抗肿瘤治疗的依从性。所有晚期肺癌患者都应全程接受姑息医学的症状筛查、评估和治疗。筛查的症状既包括疼痛、呼吸困难、乏力等常见躯体症状，也应包括睡眠障碍、焦虑抑郁等心理问题。

生活质量评价应纳入晚期肺癌患者的整体评价体系和姑息治疗的疗效评价中。推荐采用欧洲癌症研究与治疗组织生活质量测定量表(European Organzation for Research and Treatment of Cancer quality of life-C30, EORTC QLQ-C30)(V3.0)中文版进行整体评估，还可采用生命质量测定量表EORTC QLQ-LC13筛查和评估晚期肺癌患者的常见症状。

疼痛和呼吸困难是影响晚期肺癌患者生活质量的最常见症状。

1、疼痛

(1)、评估

患者的主诉是疼痛评估的金标准，镇痛治疗前必须评估患者的疼痛强度。疼痛评估首选数字疼痛分级法，有认知障碍的老年人可用脸谱法。疼痛强度分为3度，即轻度、中度和重度；不仅要记录患者评估当时的疼痛强度，还要了解过去24 h以内的最重、最轻和平均疼痛强度，了解静息和活动状态下的疼痛强度变化。

评估内容包括疼痛的病因、特点、性质、加重或缓解因素、疼痛对患者日常生活的影响、镇痛治疗的疗效和副作用等。推荐采用简明疼痛量表进行评估。

评估时还要明确患者是否存在肿瘤急症所致的疼痛，以便立即进行有关治疗。常见的肿瘤急症包括：病理性骨折或承重骨的先兆骨折；脑实质、硬脑膜或软脑膜转移癌；与感染相关的疼痛；内脏梗阻或穿孔等。

(2)、治疗

疼痛治疗的目标是使疼痛强度降至轻度以下，甚至无痛，同时要尽可能实现镇痛效果和副作用间的最佳平衡。WHO按阶梯镇痛原则仍是临床镇痛治疗应遵循的最基本原则，阿片类药物是癌痛治疗的基石，对乙酰氨基酚和非甾体类抗炎镇痛药物是重要的辅助镇痛药物。80%以上的癌痛可通过药物治疗得以缓解，少数患者需非药物镇痛手段，包括外科手术、放疗止痛、微创介入治疗等，应动态评估镇痛效果，积极开展学科间的协作。

(3)、患者及其亲属的宣教

应告诉患者及亲属镇痛治疗是肿瘤整体治疗的重要内容，忍痛对患者百害无益。吗啡及其同类药物是癌痛治疗的常用药物，罕见成瘾；要在医务人员指导下进行镇痛治疗，患者不能自行调整治疗方案和药物剂量；要密切观察疗效和药物的副作用，随时与医务人员沟通，定期复诊。

2、呼吸困难

呼吸困难是晚期肺癌患者最常见的症状之一。晚期肺癌患者中70%可伴有呼吸困难，肺癌患者死亡前90%存在呼吸困难。

呼吸困难是主观的呼吸不适感，患者的主诉是诊断的金标准。呼吸困难的临床表现为呼吸频率、节律和幅度的改变，严重者还有濒死感，恐惧和焦虑均会加重呼吸困难。

应充分认识到晚期肺癌患者呼吸困难的复杂性，尽可能祛除可逆病因。可有针对性地给予抗肿瘤、抗感染治疗；慢性阻塞性肺部疾病给予支气管扩张剂、糖皮质激素；上腔静脉和支气管阻塞者应用糖皮质激素、放疗或置人支架等；胸腔积液时给予胸腔穿刺引流术等。

阿片类药物是治疗癌症患者呼吸困难的最常用药物。及早给予阿片类药物，能减少患者的生理和心理负担，延长生存期。吗啡是首选药物，治疗呼吸困难时的使用方法与镇痛治疗一致。建议小剂量起始，按时给药，缓慢增量，严密观察和防治副作用。老年患者的增量更应谨慎。镇静剂是阿片以外的有效药物，有助于缓解急性或重度呼吸困难。

(六)、主要特殊转移部位的治疗

1、脑转移的治疗

肺癌最常见的远处转移部位之一是脑部，20%-65%的肺癌患者会发生脑部转移，是脑转移性肿瘤中最常见的类型^[54-56]^。肺癌脑转移患者预后差，自然平均生存时间仅1个月-2个月^[57]^。目前的治疗方式主要有手术、WBRT、SRS、化疗以及分子靶向治疗。

(1)、手术治疗

手术切除肿瘤可解除肿瘤对脑组织压迫，降低颅内压，从而缓解患者症状，提高生活质量，为放化疗创造条件，延长生存期。尤其是占位效应明显、引起颅内压增高或梗阻性脑积水的单发NSCLC脑转移患者可以从手术中获益。手术治疗适用于下列患者：颅内为孤立性病灶或相互靠近的多个病灶；病灶位置较表浅、位于非重要功能区；患者的全身状态良好；肺部病灶控制良好，无其他远处转移灶。

(2)、放射治疗

WBRT可以缓解晚期肺癌脑转移患者的神经系统症状，改善肿瘤局部控制情况。WBRT用于单发病灶的术后放疗、不宜手术的单个病灶的放疗、多发病灶的放疗的患者等。虽然WBRT在一定程度上提高了DCR，治疗总有效率为60%-80%，但患者中位生存期仅为3个月-6个月^[58]^。

SRS具有定位精确、剂量集中、损伤相对较小等优点，能够很好地保护周围正常组织，控制局部肿瘤进展，缓解神经系统症状，逐渐成为脑转移瘤的重要治疗手段。SRS适用于转移瘤直径 < 3 cm、数目较少、位置较深及全身情况差不适合手术的患者，可与WBRT联合应用。

(3)、化疗

化疗是NSCLC脑转移不可或缺的治疗手段。以铂类药物为基础，联合培美曲塞、长春瑞滨等药物可给NSCLC脑转移患者带来生存获益^[59-62]^。

(4)、分子靶向治疗

分子靶向药物为NSCLC脑转移提供了新的治疗选择。对于*EGFR*基因敏感突变的NSCLC脑转移患者，EGFR-TKIs治疗的客观缓解率较高^[63-67]^。EGFR-TKIs联合WBRT治疗NSCLC脑转移患者具有一定疗效^[68, 69]^，*EGFR*基因敏感突变的NSCLC患者出现脑转移时可使用EGFR-TKIs治疗。

2、骨转移的治疗

肺癌骨转移可引起骨痛、骨痛加剧或出现新的骨痛、病理性骨折、椎体压缩或变形、脊髓压迫、因骨痛或防治病理性骨折或脊髓压迫进行的骨放疗、骨转移病灶进展及高钙血症等骨相关事件(skeletal related events, SRE)的发生，严重影响患者的生活质量，预示患者生存期缩短，肺癌骨转移后患者的中位生存时间仅6个月-10个月^[70]^。肺癌骨转移应采用以全身治疗为主的综合治疗，包括化疗、分子靶向治疗、手术、放疗和双膦酸盐治疗。在控制原发疾病的同时，积极预防和治疗SRE尤为重要。合理的局部治疗可以更好地控制SRE，双膦酸盐可以预防和延缓SRE的发生。

(1)、放射治疗

放射治疗能够减轻或消除骨痛症状、预防病理性骨折和脊髓压迫的发生、缓解脊髓压迫症状并改善患者生活质量。放射治疗适用于有疼痛症状的全身各处骨转移灶，以缓解疼痛并恢复功能，出现椎体转移有脊髓压迫时首选放疗。姑息性放疗可用于脊柱或股骨等负重部位发生的骨转移的治疗^[71]^，治疗剂量通常为Dt 30 Gy/10次。每次3 Gy。

(2)、外科治疗

手术可缓解肺癌骨转移患者痛、防止或固定骨折、恢复或维持肢体的运动功能、减少或避免运动系统功能受损或脊髓压迫症所引发的并发症并提高生活质量。对于诊断不明患者亦可通过手术获得骨转移病灶的组织学诊断。

(3)、双膦酸盐治疗

双膦酸盐是治疗肺癌骨转移的基础用药，可以和化疗、靶向治疗、放疗和外科治疗联合使用。肺癌患者明确诊断骨转移后，如无双膦酸盐应用禁忌症，均推荐应用双膦酸盐治疗。第一代双膦酸盐药物(羟乙膦酸、氯膦酸)、第二代双膦酸盐药物(帕米膦酸)及第三代双膦酸盐药物(伊班膦酸钠、唑来磷酸)均能改善肺癌骨转移患者的疼痛、控制病情、预防和延缓SRE的发生并提高患者生活质量。

八、晚期肺癌患者的随访

新发晚期肺癌患者诊治后应定期随访并进行相应的检查。检查方法包括病史、体检、血液肿瘤标志物检查、影像学检查和内镜检查等，随访频率为治疗后每3个月随访1次，根据病情变化采取相应的治疗措施。

《中国晚期原发性肺癌诊治专家共识(2016年版)》的制订参考了国际和国内权威的肺癌诊疗指南^[5, 9, 72-77]^以及国内外最新研究进展。临床工作中每位患者的病情和身体状态存在较大差异，医生需根据患者的具体情况制定个体化的治疗方案，本共识仅作参考。

**专家组成员**

**顾问**

**孙燕        中国医学科学院北京协和医学院肿瘤医院，抗肿瘤分子靶向药物临床研究北京市重点实验室**

**于金明       山东省肿瘤医院**

**专家组组长**

**石远凯       中国医学科学院北京协和医学院肿瘤医院，抗肿瘤分子靶向药物临床研究北京市重点实验室**

**委员(排名不分先后，按姓氏笔画为序)**

**丁翠敏       河北医科大学第四医院**

**王子平       北京大学肿瘤医院**

**王长利       天津医科大学附属肿瘤医院**

**王东        第三军医大学大坪医院**

**王存德       云南省肿瘤医院**

**王征        卫生部北京医院**

**王孟昭       北京协和医院**

**支修益       首都医科大学宣武医院**

**石远凯       中国医学科学院北京协和医学院肿瘤医院，抗肿瘤分子靶向药物临床研究北京市重点实验室**

**卢铀        四川大学华西医院**

**冯继锋       江苏省肿瘤医院**

**刘云鹏       中国医科大学附属第一医院**

**刘晓晴       中国人民解放军第307医院**

**刘巍        北京大学肿瘤医院**

**伍钢        华中科技大学协和医院**

**李小梅       解放军总医院**

**李凯        天津医科大学附属肿瘤医院**

**李恩孝       西安交通大学第一附属医院**

**李薇        吉林大学第一医院**

**陈公琰       哈尔滨医科大学附属肿瘤医院**

**陈正堂       第三军医大学新桥医院**

**余萍        四川省肿瘤医院**

**吴宁        中国医学科学院北京协和医学院肿瘤医院**

**吴密璐       青海大学附属医院**

**肖文华       中国人民解放军总医院第一附属医院**

**张力        北京协和医院**

**张沂平       浙江省肿瘤医院**

**张树才       首都医科大学附属北京胸科医院**

**杨树军       河南省肿瘤医院**

**宋霞        山西省肿瘤医院**

**林冬梅       北京大学肿瘤医院**

**罗荣城       南方医科大学南方医院**

**单莉        新疆医科大学肿瘤医院**

**周彩存       同济大学附属上海市肺科医院**

**周宗玫       中国医学科学院北京协和医学院肿瘤医院**

**赵琼        浙江大学附属第一医院**

**胡成平       中南大学湘雅医院**

**胡毅        中国人民解放军总医院**

**郭其森       山东省肿瘤医院**

**常建华       复旦大学附属肿瘤医院**

**黄诚        福建省肿瘤医院**

**曾瑄        北京协和医院**

**韩宝惠       上海交通大学附属胸科医院**

**韩晓红       中国医学科学院北京协和医学院肿瘤医院，抗肿瘤分子靶向药物临床研究北京市重点实验室**

**学术秘书**

**郏博        中国医学科学院北京协和医学院肿瘤医院，抗肿瘤分子靶向药物临床研究北京市重点实验室**

**韩颖        中国医学科学院北京协和医学院肿瘤医院，抗肿瘤分子靶向药物临床研究北京市重点实验室**

**黄昱        中国医学科学院北京协和医学院肿瘤医院，抗肿瘤分子靶向药物临床研究北京市重点实验室**
